# Cost‐effectiveness of an online randomised controlled trial for dementia prevention, Maintain Your Brain, to improve cognition and lower dementia risk after 3 years

**DOI:** 10.1002/alz.088790

**Published:** 2025-01-09

**Authors:** Heidi Welberry, Li‐Jung Elizabeth Ku, Sophy TF Shih, Louisa Jorm, Maria A. Fiatarone Singh, Michael Valenzuela, Jeewani Anupama Ginige, Megan Heffernan, Tiffany Chau, Henry Brodaty

**Affiliations:** ^1^ UNSW Sydney, Sydney, NSW Australia; ^2^ National Cheng Kung University, Tainan Taiwan; ^3^ University of New South Wales, Sydney, NSW Australia; ^4^ University of Sydney, Sydney, NSW Australia; ^5^ Western Sydney University, Penrith, NSW Australia; ^6^ George Institute, University of New South Wales, Sydney, NSW Australia; ^7^ Centre for Healthy Brain Ageing (CHeBA), UNSW Sydney, Sydney, NSW Australia; ^8^ University of New South Wales (UNSW), Sydney, NSW Australia; ^9^ University of New South Wales, Sydney Australia

## Abstract

**Background:**

The Maintain Your Brain (MYB) randomised controlled trial (RCT) examined the effect of a multi‐domain internet‐based dementia prevention program against a control group (information only) over three years among Australians aged 55‐77 years. A cost‐effective analysis (CEA) quantified the differences in costs (direct healthcare and program costs) and effectiveness outcomes between the intervention and control groups from a health care sector perspective.

**Method:**

An economic evaluation was conducted alongside the MYB trial. The intervention comprised a personalised schedule of online coaching in physical activity, nutrition, cognitive activity, and depression or anxiety. MYB trial data were linked to the 45 and Up Study and administrative health data using uniquely assigned identifiers to obtain healthcare utilisation and costs data. The two effectiveness outcomes were global cognition composite (GCC) scores and the Australian‐National University Alzheimer’s Disease Risk Index (ANU‐ADRI) short form questionnaire scores. Costs analysed included MYB program costs and the direct healthcare costs of MYB participants. All costs were reported on actual nominal values in Australian dollars ($AUD) during the trial period. The time horizon of this analysis was 3 years follow‐up after randomization. Incremental cost effectiveness ratio (ICERs) between the intervention and the control were calculated for GCC and ANU‐ADRI and compared using bootstrapped means and bias corrected and accelerated 95% Confidence Intervals.

**Result:**

There were 3025 in the intervention group and 3033 in the control with available linked healthcare data. The average program cost per person was $1,572 for the intervention group and $538 for the control. Incremental cost‐effectiveness ratios at three years were calculated by comparing the average marginal difference in costs to a mean difference in z score of 0.18 (95%CI: 0.13, 0.23) and in ANU‐ADRI of ‐0.57 (95%CI: ‐0.95, ‐0.24) for the intervention versus control. The base case ICERs calculated were $2568 per 1 standard deviation in Z score and $823 per reduction of 1 ADRI point. After 1000 bootstrapped replications, the scatter‐plots of ICER ellipses suggests that the MYB intervention was effective and relatively cost‐neutral.

**Conclusion:**

The MYB program showed potential cost‐effectiveness for preventing dementia. Longer‐term follow‐up is needed to confirm our initial findings.

